# Cerebellar Nuclei Neurons Show Only Small Excitatory Responses to Optogenetic Olivary Stimulation in Transgenic Mice: *In Vivo* and *In Vitro* Studies

**DOI:** 10.3389/fncir.2016.00021

**Published:** 2016-03-24

**Authors:** Huo Lu, Bo Yang, Dieter Jaeger

**Affiliations:** ^1^Department of Biomedical Sciences, Philadelphia College of Osteopathic Medicine—Georgia CampusSuwannee, GA, USA; ^2^Department of Biology, Emory UniversityAtlanta, GA, USA

**Keywords:** optogenetics, inferior olive, climbing fiber collaterals, slice, single unit

## Abstract

To study the olivary input to the cerebellar nuclei (CN) we used optogenetic stimulation in transgenic mice expressing channelrhodopsin-2 (ChR2) in olivary neurons. We obtained *in vivo* extracellular Purkinje cell (PC) and CN recordings in anesthetized mice while stimulating the contralateral inferior olive (IO) with a blue laser (single pulse, 10–50 ms duration). Peri-stimulus histograms (PSTHs) were constructed to show the spike rate changes after optical stimulation. Among 29 CN neurons recorded, 15 showed a decrease in spike rate of variable strength and duration, and only 1 showed a transient spiking response. These results suggest that direct olivary input to CN neurons is usually overridden by stronger PC inhibition triggered by climbing fiber responses. To further investigate the direct input from the climbing fiber collaterals we also conducted whole cell recordings in brain slices, where we used local stimulation with blue light. Due to the expression of ChR2 in PC axons as well as the IO in our transgenic line, strong inhibitory responses could be readily triggered with optical stimulation (13 of 15 neurons). After blocking this inhibition with GABAzine, only in 5 of 13 CN neurons weak excitatory responses were revealed. Therefore our *in vitro* results support the *in vivo* findings that the excitatory input to CN neurons from climbing fiber collaterals in adult mice is masked by the inhibition under normal conditions.

## Introduction

**Figure 1:**
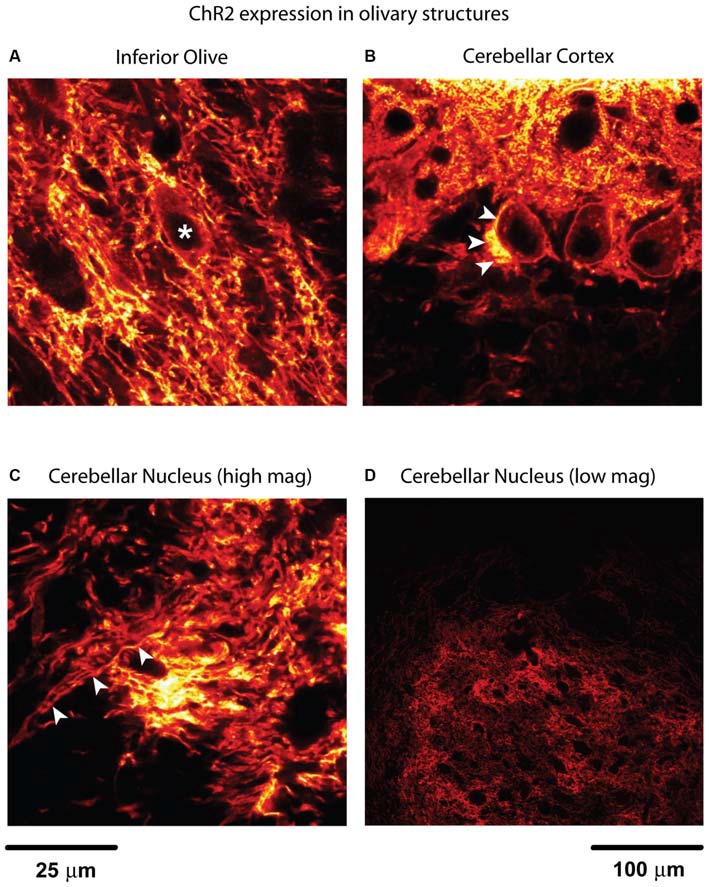
**Channelrhodopsin-2 (ChR2) expression in inferior olive (IO) and cerebellum. (A)** Fibers with expression in the IO form bundles to travel through the space in between dark spaces where the cell bodies of olivary neurons are located (*). A bright lining around cell bodies indicates membrane-bound ChR2. This is determined by the size and the shape of the structure. Putative local collateral terminals showed brighter staining than somatic membrane, however, suggesting enrichment of ChR2 in terminals. **(B)** The strongest expression in this image can be found near the centered Purkinje cell (PC) soma, which is believed to be climbing fiber (arrowheads) traveling up to synapse onto the proximal dendrite. Labeling was also observed in the perisomatic membrane of PCs and dendrites. **(C)** The image was taken from the anterior part of the cerebellar nuclei (CN) where the IO collateral fibers enter the nucleus from the cerebellar peduncle (indicated by arrowheads). These fibers may also contain PC axon fibers. ** (A–C)** share the same scale bar. **(D)** To illustrate the distribution of these axon fibers in the CN, a low magnification image from parasagittal section is used. This is the anterior interpositus nucleus which is comparable with the site shown in **(C)**. The black holes in the CN are either the lumen of blood vessels or the cell bodies of the CN neurons.

Neurons in the cerebellar nuclei (CN) are often seen as primarily processing GABAergic input from cerebellar cortical Purkinje cells (PCs) to provide the final output from the cerebellum. Nevertheless, CN neurons also receive a substantial number of glutamatergic inputs, namely from axon collaterals of the mossy fibers (Chan-Palay, 1977; Wu et al., 1999) and axon collaterals from the inferior olive (IO; Wiklund et al., 1984; Van der Want et al., 1989; Ruigrok and Voogd, 2000) that branch off the climbing fibers that powerfully excite PCs (Eccles et al., 1966; Llinás and Sugimori, 1982). While the role of mossy fiber input to the CN has been integrated in functional models of the cerebellum (Holdefer et al., 2005; Ohyama et al., 2006; Thompson and Steinmetz, 2009), no such functional role has been assigned to the climbing fiber collaterals to date. Anatomically it is known that climbing fiber collaterals from the IO result in a plexus of fine terminals in the rat CN (Van der Want et al., 1989), but even the most basic synaptic properties such as postsynaptic current amplitudes and time courses have not been determined. This is primarily because olivary input to the CN cannot be selectively stimulated with traditional electrical stimulation methods in brain slices, and no distinguishing pharmacological features of synaptic transmission in this pathway have been described. In the present study, we used optogenetic techniques to selectively stimulate the climbing fiber collateral input to CN neurons to characterize this potentially important cerebellar pathway *in vitro* and *in vivo*. Channelrhodopsin-2 (ChR2) expression in the IO was achieved with the Ptf1a gene as a Cre driver and crossing Ptf1a-Cre mice to a Rosa Ai27 ChR2-tdTomato reporter line as previously described (Kawaguchi et al., 2002; Renier et al., 2010; Zhu et al., 2011). ChR2 expression was absent in other brain areas except in cerebellar PCs (Figure 1). We studied the consequences of olivary input to CN neurons *in vivo* with optical stimulation directly onto the IO in anesthetized mice. In addition, we characterized the synaptic properties of olivary input to CN neurons in brain slices by stimulating climbing fiber collaterals.

## Materials and Methods

### Transgenic Mice

Transgenic mice with Cre dependent ChR2 expression in the IO were obtained by crossing heterozygous Ptf1a-Cre mice with a homozygous Ai27 ChR2-td Tomato (Rosa) line and determining successful crosses with genotyping, which was performed by Transnetyx, Inc. from tail snips. The heterozygous Ptf1a-Cre strain was obtained from Chris De Zeeuw’s lab in Rotterdam and regenerated in the animal facility at Emory University on a C57BL/6J background. A heterozygous Ai27 Rosa strain was also obtained from the De Zeeuw lab, rederived on a C57BL/6J background at Emory University, and bred to homozygocity. All protocols were approved by the IACUC of Emory University and complied with the NIH guide on animal use.

### Surgery for *In Vivo* Recordings

Two to six-month old male transgenic mice were anesthetized with isoflurane in an induction chamber (4% isoflurane in 100% oxygen at 1 L/min) and 17 of 22 mice were injected with urethane (30%, 0.2 ml i.p.), before being moved to a mouse stereotaxic apparatus, where they continually received isoflurane from a nose cone (2% with oxygen at 0.6 L/min). After the surgery, the oxygen flow was kept the same through the rest of the experiment with zero isoflurane while animals remained under urethane anesthesia (17/22 mice) or were maintained under lighter 1% isoflurane anesthesia (5/22 mice) for neural recordings. Sufficient depth of anesthesia was checked for by the absence of withdrawal reflexes. The animal’s body temperature was monitored using a mouse rectal probe and maintained at 36^°^C by feedback through a heating blanket (FHC Inc., Bowdoin, ME, USA). The skin over the cerebellum and brainstem was incised, and the muscles over the brainstem were separated from the midline to expose the foramen magnum. After a small cut of the dura, the obex was visible and used as a reference point. An optic fiber (62 μm or 200 μm diameter; GIF625-CUSTOM or BFH37-200-CUSTOM, Thorlabs, Inc.) was lowered to the target coordinates of the IO with a 20^°^C angle from the coronal plane. The light stimulation site was 0.5 mm lateral from the midline and 2.5 mm in depth from the surface of the brainstem. Light stimulation was performed with a blue laser (SDL-473-060T, Shanghai Dream Laser) and consisted of single light pulses of 10 or 50 ms duration. The blue laser (wavelength: 473 nm) was at 1.26 mW while exiting the fiberoptic. A small craniotomy was prepared in the skull above the cerebellar hemisphere contralateral to the side of light stimulation. The dura was removed to expose the crus I and II regions. The exposed surface was covered by physiological saline.

### Extracellular Recordings

Recordings were obtained with 1.5 mm OD glass pipettes pulled with a horizontal puller (P-97, Sutter Instrument) and broken to a tip size of 5–10 μm under a microscope. Electrodes were filled with pontamine sky blue (1 M in saline solution), mounted on a motorized manipulator (MP-285, Sutter Instrument, Novato, CA, USA) and inserted above the cerebellar cortex and the CN. Once a neuron was isolated in the cerebellum, a single light pulses were delivered to the contralateral IO to stimulate climbing fiber inputs. Single unit responses were recorded first (filter setting: 300 Hz–10 kHz), followed by local field potential (LFP) responses with the low pass filter opened to 1 Hz. At the end of a successful recording from a neuron at the depth of the CN, 10 μA of continuous current was applied for 10 min to have the blue dye ejected at the recording site. Upon successful dye deposition, the recording site could be seen as a blue dot on the tissue sections following histological recovery.

### Slice Preparation

In order to obtain good slice quality from adult transgenic mice (2–6 months), we perfused deeply anesthetized animals (ketamine 100 mg/kg, xylazine 20 mg/kg, i.p. injection) with a sodium-replaced ice-cold solution (in mM: Choline Chloride 117.7, KCl 2.5, NaHPO_4_, 1.25, MgCl_2_ 7, NaHCO_3_ 26, D-Glucose 10, CaCl_2_ 0.5, L-Ascorbate 1, Sodium Pyruvate 3, saturated with 95% O_2_ and 5% CO_2_) prior to slicing. The slice preparation procedure follows published protocols (Koos and Tepper, 2002; Tecuapetla et al., 2005). Briefly, after decapitation, the cerebellum was quickly removed and was immediately immersed in ice-cold artificial cerebrospinal fluid (ACSF) containing (in mM: NaCl 124, KCl 2.5, NaH_2_PO_4_ 1.25, MgCl_2_ 1.3, NaHCO_3_ 26, D-glucose 10, CaCl_2_ 2, L –Ascorbate 1, Sodium pyruvate 3), bubbled with 95% O_2_ and 5% CO_2_. Then, a small block of cerebellum was mounted for sectioning with a vibratome (Thermo Scientific hm650 V, MA, USA). Two to three sagittal slices (250 μm in thickness) containing the CN were obtained and allowed to recover for 1 h at 34^°^C and then continually incubated at room temperature until use.

### Whole Cell Patch Clamp Recordings

Slices were transferred to a submerged-type chamber mounted on a Zeiss Axioscope fixed stage microscope with differential interference contrast (DIC) optics. Slices were superfused with ACSF (gassed with 95% O_2_ and 5% CO_2_) throughout the recording at a flow rate of 1.5 – 2 ml/min at 32^°^C. The recording temperature was maintained by a Peltier slice chamber heating element (Luigs and Neumann, Ratingen, Germany). Borosilicate glass-patch electrodes (1.5 mm OD) were pulled on a Sutter P-97 horizontal puller, and filled with intracellular pipette solution containing (in mM): K-gluconate 140, HEPES 10, NaCl 6, EGTA 0.2, MgATP 4, NaGTP 0.4, spermine 0.05, glutathione 5. The final electrode resistance was between 4 and 7 MΩ. Neurons in the CN were visualized with a 60× objective, the pipette lowered using a Luigs and Neumann manipulator, and pipette tips located over a neural membrane before seal formation was attempted with suction under manual control. After break-in, whole cell patch-clamp recordings in either voltage-clamp or current clamp mode were acquired and controlled using an Axon 700 B Multiclamp amplifier (Molecular Devices, Sunnyvale, CA, USA). Recordings were sampled at 10 kHz using an NI PCI-6052E DAQ card (National Instruments, TX, USA) and Labview 8 (National Instruments, TX, USA) was used to apply current injection pulse paradigms during recordings.

Light stimulation during brain slice recordings was performed with 5 to 10 ms full-field flashes controlled by a Uniblitz shutter (Vincent Associates, Rochester, NY, USA) from a 100 W microscope mercury bulb using a FITC filter set to selectively pass blue light. The blue light cover an area of circle in a diameter of 0.8 to 1 mm, which is about the size of CN in a sagittal section. The optical power is 5.1 mW measured at the slice level for the light generated by the mercury lamp filtered by a narrow band filter passing light around 470 nm. This level of light is more than sufficient for a maximal response.

At the beginning of each recording, a series of 1 s hyperpolarizing or depolarizing current steps was applied in current clamp to check the quality of the recording by spike size (>60 mV) and access resistance (>25 MΩ). EPSCs were recorded under voltage clamp while the cells were held at −60 mV).

### Histology

At the end of *in vivo* experiments, animals were perfused transcardially with a solution of 15% sucrose in 10% phosphate buffered formalin. The brain was removed and saved in the same fixative with 30% sucrose. Tissue was sectioned sagittally in 50 μm slices using a freezing microtome and sequentially mounted onto microscope slides. ChR2 expression specificity was verified under a confocal microscope (SP8, Leica Microsystems, Germany) by identifying the neuronal structures labeled by the presence of the tdTomato fluorescent protein (Figure 1). Since the possibility of expression of ChR2 in mossy fibers would affect the results, the expression was examined carefully, no axon fibers terminate in the granule cell layer matched the features of mossy fibers. Only those fibers traveling straight in and out as PC axons and climbing fibers were observed. This is in consistent with the early studies of the involvement of Ptf1a in climbing fiber neuron development (Yamada et al., 2007).

### Data Analysis

Data from both *in vivo* and *in vitro* experiments was saved in hierarchical data format release 5 (HDF5) using customized software written in Labview 8.0 (National Instruments, Inc.). All data analysis was performed in Matlab (The Mathworks, Inc., Natick, MA, USA). For the spike detection, a threshold was normally set at three times of the standard deviation calculated from the spike recording traces. The timing of the maximum value will be used for the calculation of the spike rate. To examine the spike rate changes in response to the light stimulation, spikes were convolved with a Gaussian kernel (sigma = 5 ms). The Gaussians of all spikes were summed and normalized to represent a slightly smoothed instantaneous spike rate (Rowland and Jaeger, 2005). To determine responses to optogenetic stimuli, the averaged spike rate in 1 s before the stimulus was used as the baseline, and if the spike rate within a 100 ms window after the stimulus passed three times the standard deviation of the baseline, we counted this rate change as a response. The response amplitude was measured as the change of the spike rate from the baseline to the peak value of the event. The response duration was measured where the rising and falling phase of the event crossed the baseline rate.

## Results

### LFP Responses to the Optogenetic Stimulations in the IO *In Vivo*

**Figure 2:**
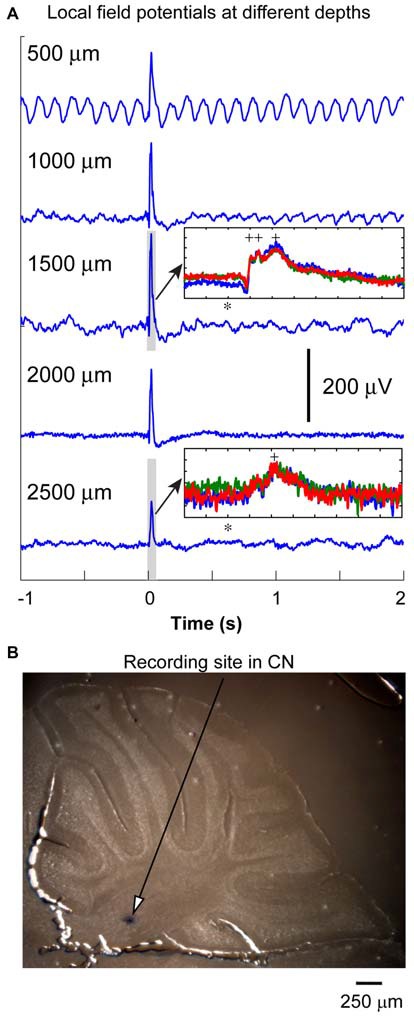
**Local field potential (LFP) recording from cerebellum. (A)** LFP responses to IO optical stimulation were recorded at different depths at 500 μm intervals. The oscillation in the first trace was from the pulsation due to the heart beat. Optic stimulation (10 ms duration) was delivered at the time denoted with zero to the IO to evoke these responses. The amplitude of the LFP responses reached the maximum at the depth about 1500 μm. The LFP responses normally contained multiple peaks (inset, 100 ms window represents the duration of gray box, stimulation time is represented by *, peaks are labeled by +). It became weaker at the depth of the CN where a neuron can be isolated (see inset for LFPs at the depth of 2500 μm). Note: scale for *y* axis of the two insets are different, each division is 50 μV. **(B)** After a successful recording, blue dye is injected to label the recording site in the CN. In this specific penetration (different from the one having LFP responses recorded), the blue dot was found to be in anterior interpositus nucleus at the depth of 2400 μm.

Twenty two transgenic mice were used in this part of the experiments. In some of the penetrations (*n* = 13), we recorded LFP responses to optical stimulation every 500 μm along the electrode track to obtain a depth profile of the cerebellar responses to the olivary inputs. As shown in Figure 2, the LFP responses increased in amplitude at a depth between 1000 and 2000 μm, and then decreased. This depth profile was similar in all other recordings. The depth for the CN was in between 2000 and 2500 μm (Figure 2B, blue dot). In 13 out of 22 animals, the locations of the blue dots were found to confirm recording sites in the CN. The peaks of LFP responses in the CN occurred between 20 to 30 ms from the stimulation time. In some of the experiments (*n* = 13), LFP responses recorded in the cerebellar cortex (depth < 1500 μm) contained multiple peaks (inset in Figure 2, 100 ms window, three traces are demonstrated). The earlier peaks (first two labeled by +, 10 to 20 ms from stimulation) were not significant for the responses recorded from the CN regions. This suggests that these LFP events, especially those recorded from cerebellar cortical regions represent a population climbing fiber responses to the optogenetic stimulation. Since the LFP recorded from the CN region (at the depth between 2000 and 2500 μm) contained only the later event with a lower amplitude (see inset in Figure 2), this response may represent a delayed PC input to the CN. Therefore, an early LFP peak corresponding to the direct excitation of CN neurons at the time that PCs showed such a response, was not as significant as those in the first inset. This suggests that the direct input from the olivary fibers to CN neurons may not generate a strong population EPSP, which would likely result in a noticeable extracellular LFP response.

### The Spike Rate of CN Neurons Decreased following Olivary Stimulation in Most of the Recordings

**Figure 3:**
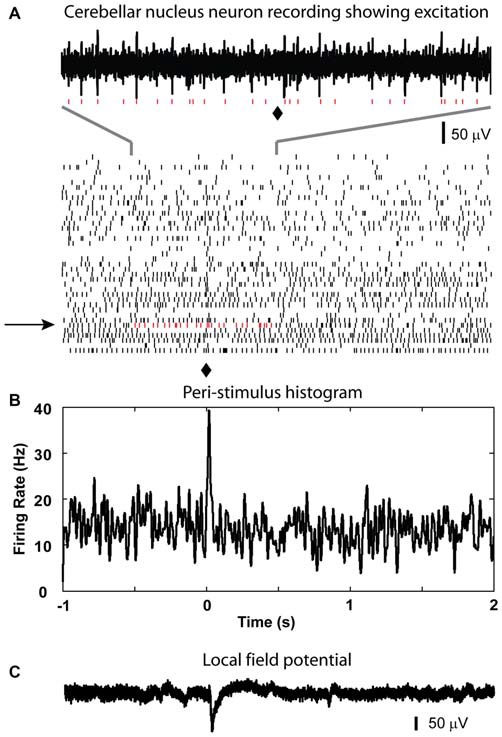
**Excitatory response from CN neuron to optical stimulation in the IO. (A)** Raster plot generated using the spikes recorded from a CN neuron at the depth of 2267 μm (40 sample trials, corresponding trial of the example trace is indicated by black arrow, red dots are used to show detected spikes in the example trace). **(B)** A peri-stimulus histogram (PSTH) plot is constructed to show the response to the light stimulation given at 0 s in the IO (⧫ in raster plot). It demonstrated a weak transient increase in firing rate after the light stimulation. **(C)** LFP recorded at the same site of the isolated cell.

**Figure 4:**
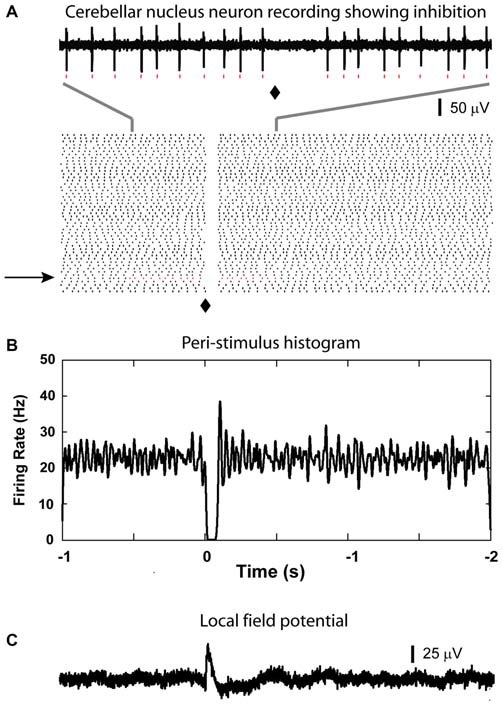
**Inhibitory responses from CN neuron. (A)** A clear suppression of spiking is visible immediately after the light stimulation (⧫) in the raster plot (based on 50 sample traces, raw data in the top panel is generated from the trail indicated by the black arrow, red dots can be matched for the individual spikes). **(B)** Corresponding to the suppression shown in the raster plot, there is a 100 ms time window in the PSTH where firing was reduced to zero in this CN neuron. Note that there was a slight increase in firing rate after the main inhibitory event. **(C)** LFP recorded from the same region of this isolated cell.

After recording LFP traces, single units were isolated below 2000 μm depth to record CN spike activity when possible. We recorded 29 CN neurons. These neurons showed baseline firing rates ranging from 4 to 64 Hz (mean rate = 33.7 ± 3.2 Hz). Only 1 neuron showed a statistically significant increase in spike rate following olivary stimulation. This response consisted mostly of a single added spike to the background activity (Figure 3). Thirteen neurons showed no responses to the stimulation based on our rate change requirements. The remaining 15 cells showed a decrease in spike rate of variable strength and duration (latency = 10.2 ± 1.9 ms; duration = 71.0 ± 10.0 ms; Figure 4). The average decrease of firing rate following olivary stimulation was 31.3 ± 3.3 Hz measured from the baseline to the trough of the inhibition. For 4 of these 15 CN neurons the rate dropped to zero. The duration of the decrease in firing rate varied from 27 to 168 ms. Four out of the 15 inhibited CN neurons showed an increase in firing rate after the inhibitory response. This response was identified by the increase in firing rate above three times of the standard deviation from the baseline firing rate prior to stimulation. This response had a delay ranging from 0.083 to 0.97 s and a firing rate increase from 14.28 to 32.05 Hz.

### Olivary Input to Purkinje cells

**Figure 5:**
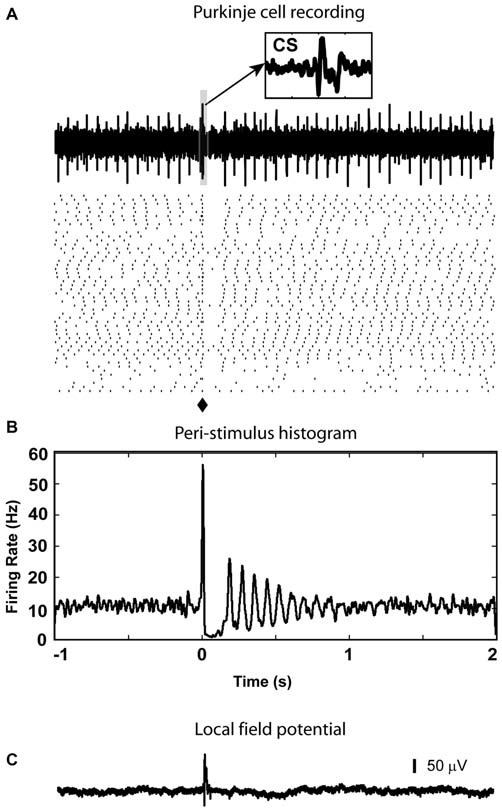
**PC complex spike response to olivary stimulation. (A)** Spikes triggered in cerebellar cortex by light stimulation in the IO are complex spikes (CS) followed by a gap with very few simple spikes in the raster plot. Notice that in the sample trace, there is a complex spike (CS) which is evoked by the light stimulation (⧫). A 20 ms window (highlighted by the light blue box) starting from stimulation time is enlarged to be an inset to show waveform of the CS (arrow). **(B)** In the PSTH, the spike pause caused by the CS is reflected by a decrease in firing rate which lasted about 200 ms. **(C)** At the same depth (877 μm from the surface), LFP responses were recorded (delay = 10 ms, duration = 23 ms).

In some animals we recorded PCs (*n* = 15) in the regions of Crus I or II in the same tracks that at a greater depth targeted the nuclei. When there was a response (7 neurons), complex spikes (CS) could be detected between 7 to 10 ms after stimulation as a stereotypic multi-peaked waveform in the high frequency single unit signal (Figure 5A) as well as a positive deflection in the low frequency LFP signal (Figure 5C), and was followed by a 50 to 200 ms window without spikes in 5 neurons (Figures 5A,B). The remaining 8 neurons without CF response also showed no significant responses in simple spikes.

### Optical Stimulation of Olivary Input Induced Excitatory Postsynaptic Responses in CN Neurons *In Vitro*

**Figure 6:**
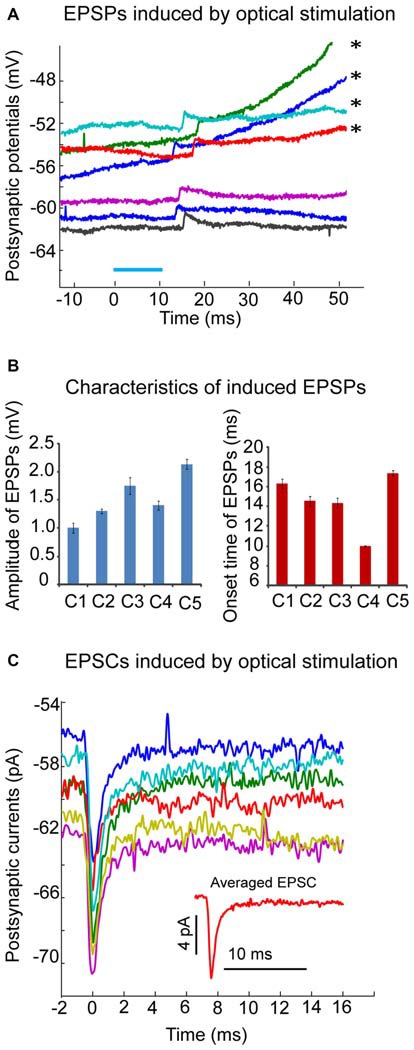
**Excitatory postsynaptic responses induced by optical stimulation in CN neurons of transgenic mice. (A)** Example of neuron where local optical stimulation (blue light, 10 ms, blue bar), elicits a small EPSP with an average amplitude of 1.8 mV in the presence of GABAzine (10 μM) under current clamp. On the top few traces marked by asterisks, the EPSPs are able to trigger action potentials at a variable delay (not shown); **(B)** Amplitudes and onset time of optical stimuli-induced EPSPs from 5 individual neurons were plotted in two bar graphs. Note the varying onset time of induced EPSPs; **(C)** The averaged EPSCs (from 6 trials) induced by optical stimulation in the same CN neuron as the one in panel A under voltage clamp (holding potential is −60 mV). Note the small amplitude (about 7 pA) of the averaged trace of EPSCs (shown in the inset figure). EPSCs were aligned to their peaks at time 0.

Forty adult (3–6 month old) transgenic Ptf1a-CRE-Rosa mice were used in this study to examine the postsynaptic responses of CN neurons to the activation of olivary input in cerebellar slices. The following criteria were used to accept recordings: (a) recorded neurons were located in one of the three (medial, lateral or interpositus N) CN regions; (b) the amplitude of spikes was larger than 60 mV; and (c) the series resistance did not exceed 30 MΩ. Fifteen recordings fulfilled these criteria. The average spike width was 0.39 ± 0.06 ms. Local optical stimulation with blue light in the CN elicited a prominent inhibitory postsynaptic potential (IPSP) or current (IPSC) in 13 neurons (data not shown; medial N:7; lateral N:6). As a large proportion of PCs expressed ChR2 in our Pt1fa-CRE-Rosa mouse crosses, these events are expected from direct stimulation of PC inputs. The IPSP/IPSC could be completely abolished by application of GABAzine at 10 μM. In the presence of GABAzine, excitatory postsynaptic potentials (EPSPs) or currents (EPSCs) were revealed in 5 of these 13 neurons (Figure 6). The amplitude of the EPSP was small (Figure 6A), with averaged EPSP amplitude ranging from 1.0 to 2.14 mV (Figure 6B), suggesting a weak excitatory input from olivary fibers in these neurons. Accordingly, EPSCs with small amplitude were seen in voltage clamp (Figure 6C). Another interesting characteristic of the elicited EPSPs or EPSCs was the different onset time in each individual trial, varying from 10 to 20 ms following the optical stimulus, which may be due to delayed activation of spikes in stimulated fibers with low ChR2 density. Taken together, our *in vitro* results support the conclusion that the excitatory input to the CN neurons from climbing fiber collaterals is weak in adult mice.

## Discussion

### Response of CN Neurons to the Light Stimulation of IO is Weak in Adult Mice

In this study, we used optogenetic stimulation to evoke the excitatory input directly from the IO without contamination of input from mossy fibers or antidromic stimulation, which might readily occur with electrical stimulation in the olive. As olivary axons reach the cerebellum, they give off collaterals to the CN neurons before entering the cerebellar cortex (Kitai et al., 1977). Therefore, in our *in vivo* recordings we expected to see excitatory events in CN recordings before PC inhibition driven by the CS from climbing fibers. However, contrary to expectations, our *in vivo* CN recordings showed only rare and weak excitatory response despite reliable later inhibition (Figure 4). Indeed a population LFP response in the CN at the time of expected direct olivary excitation was also absent. This finding suggests that the direct olivary excitatory drive is weak. A potential confound to this conclusion may be the lack of adequate ChR2 expression in olivary neurons to trigger action potentials with optical stimulation, or indeed a poorly placed stimulation location. However, the same stimulation conditions that failed to produce reliable excitation did produce inhibition at a latency following cerebellar cortical complex spike responses in most cases, indicating reliable activation of olivary efferents to the cerebellum. Another concern is the effect of anesthesia. The dose of urethane used in this study is 2 g/kg (i.p.), which is close to the dose used in previous publication from our laboratory (Lobb and Jaeger, 2015). The plasma concentration of urethane administered at this dose is equal or lower than 0.67 mM. During surgical anesthesia in mammals are estimated to be equal or larger than 10 mM. At concentration of 10 mM, urethane has modest effects on all inhibitory or excitatory neurotransmitter-gated channels (Hara and Harris, 2002). Therefore, the dose we used in our study has very little effect on the responses using ionotropic glutamatergic transmission. This dose of urethane has also been used in other *in vivo* experiments (Takahashi et al., 2006; Kitaura et al., 2010).

### Inhibition of CN Neurons by Olivary Activation Vastly Exceeds Excitatory Responses

The same optical olivary stimuli that failed to demonstrate direct excitation of CN neurons did lead to a reliable LFP response throughout the cerebellum, CS responses in a subpopulation of PCs, and indeed reliable inhibitory responses of CN neurons. These inhibitory responses had the appropriate latency to reflect CS input from PCs. Therefore, a population of synchronous spike events triggered in the IO showed a net inhibition of CN neurons in our experiments. Unfortunately we were not able to discriminate between different cell populations in the CN, and therefore cannot state whether GABAergic neurons in the CN projecting back to the IO are similarly affected as excitatory projection neurons. If this was the case, the olivary stimulation would result in a momentary reduction in drive to motor output structures, while leading to further disinhibition of the IO.

CN responses to electrical stimulation in the IO have been carried out in cats (Armstrong et al., 1973; Kitai et al., 1977; Ruigrok, 1997), rats (Rowland and Jaeger, 2008), and mice (Hoebeek et al., 2010). Consistently, these studies find a weak early excitation indicated by a single additional spike followed by a pronounced inhibition and a late long-latency excitation. Similar to our results reported here only a subpopulation of CN neurons showed the early excitation, and a study in mice quantifying this proportion found 31 of 66 (47%) units with a short latency response (Hoebeek et al., 2010). In contrast, the subsequent inhibition was robustly observed in all recordings. In a different and quite elegant approach, CN response to climbing fiber input were analyzed through correlation analysis of simultaneous multi—PC CF response recordings and CN single unit recordings in the rat (Blenkinsop and Lang, 2011). Out of 100 positive complex spike—CN unit response correlations they found purely inhibitory CN responses in 70 cases, short-latency excitation followed by inhibition in 24 cases, and weak short-latency excitation alone in six cases. This outcome is in fact quite similar to the proportion of five excitatory responses in 20 overall responsive CN neurons observed in our study. The somewhat larger proportion observed by Hoebeek et al. (2010) could be explained by a small amount of direct mossy fiber stimulation.

### Slice Recordings Substantiate the Lack of a Strong Direct Excitatory Drive of Olivary Collaterals Onto CN Neurons

The finding of weak olivary excitatory input to the CN was further substantiated in our *in vitro* recordings. Here, stimulation was performed locally to recorded neurons, ensuring activation of all olivary fibers with sufficient ChR2 expression to trigger action potentials. Nevertheless, an excitatory response was only elicited in 5 of 15 recorded neurons, and this response was small in all cases. While we cannot assess the number of olivary fibers that were stimulated, we did note a strong IPSP stemming from direct PC axon stimulation in 12 of 15 recordings. This finding suggests that at least PCs expressing Pt1fa were induced to express sufficient ChR2 to trigger action potentials in their terminal zones in the CN. Combined with the *in vivo* finding that olivary stimulation reliably elicited LFP responses in the cerebellum, these findings strongly support the overall conclusion that direct olivary excitation of CN neurons is weak. Nevertheless, it is likely that not all olivary fibers express sufficient ChR2 to be stimulated and hence somewhat larger olivary inputs than observed in this study may be present. If not direct excitation, then what could be the function of olivary input to the CN? As discussed in more detail below, we see two major alternatives, namely a developmental role in guiding microzone alignment or a plasticity role in guiding long term potentiation (LTP)/long term depression (LTD) of PC and/or mossy fiber inputs to CN neurons. We cannot provide direct evidence in favor of one or the other of these alternate hypotheses in the present study, however.

### Climbing Fiber Collaterals may Provide Developmental Guidance to Align Purkinje Cell Axons to form Microcircuits in the Targeted Microzone

The immature olivary input to the cerebellum is bilateral (Bower and Payne, 1987; López-Román and Armengol, 1994) and the maturation of olivocerebellar topography involves removal of developmental paths to define laterality plus synapse elimination within largely predefined parasagittal zones (Fournier et al., 2005). These findings indicate that the olivocerebellar circuits are highly plastic with multiple routes at the beginning of the development. In the mature cerebellum, a remarkably precise topography has been established in the deep CN such that olivocerebellar microzones between cerebellar cortex and the CN are precisely aligned (Ruigrok and Voogd, 2000; Pijpers et al., 2005). Even on the single cell level CN neurons receive convergent input from climbing fiber collaterals and PCs that are innervated by the same climbing fiber (Blenkinsop and Lang, 2011). These findings suggest that physiological mechanisms of ensuring such a precise alignment are required. A potential role for climbing fiber input to the CN during development could therefore be to enable the alignment of microzones by a correlative plasticity mechanism that favors PC synapses on CN neurons that are co-activated by the same olivary neurons. A weaker olivary input in mature animals might relate to a process of maintaining alignment of maps without requiring significant spike excitation of CN neurons by climbing fiber collaterals. Our data, however, provide no specific evidence for this hypothesis, which would need to be directly tested in developmental studies.

### Contribution of Climbing Fiber Collaterals in Cerebellar Plasticity

Other than the possible guidance function in the development of cerebellar microcircuits, climbing fiber collaterals may also be important in the functional synaptic plasticity of mossy and PC inputs to CN neurons. Multiple forms of plasticity have been described in the CN that may subserve both homeostatic and learning mechanisms (Zheng and Raman, 2010). PC inputs undergo LTD with tetanic stimulation (Morishita and Sastry, 1996), as do mossy fiber inputs with burst stimulation (Zhang and Linden, 2006). In contrast, LTP of the mossy fiber input occurs in response to a sequence of excitation, inhibition and disinhibition (Pugh and Raman, 2008; Person and Raman, 2010). Interestingly, this time course of events in particular would be expected with a synchronous activation of the olivocerebellar inputs to a given microzone (Blenkinsop and Lang, 2011). The potential contribution of olivary inputs to the CN in facilitating this or other plasticity mechanism remains unknown. In fact, a characterization of olivary synapses in the CN with respect to the presence of NMDA and metabotropic glutamate receptors has not been performed to our knowledge, and the activation of such receptors would be more likely to contribute to plasticity mechanisms than the weak direct excitation of CN neurons observed in the present study. A participation of the olivary input to the CN in functional plasticity would certainly be of importance, as it would likely complement and enhance the mossy fiber LTD function that has been described for climbing fiber input to PCs for a long time (Ito, 1989).

## Author Contributions

HL did the work for the *in vivo* part. BY did the *in vitro* part. DJ is the PI for the whole project. All the authors contributed to the data analysis, manuscript preparation.

## Conflict of Interest Statement

The authors declare that the research was conducted in the absence of any commercial or financial relationships that could be construed as a potential conflict of interest.
